# Shifting IRES versus Cap-initiated translation during homeostatic stem cell differentiation and stress

**DOI:** 10.1126/sciadv.adz7896

**Published:** 2026-05-22

**Authors:** Michael C. Mazzola, Ting Zhao, Anna Kiem, Trine A. Kristiansen, Karin Gustafsson, Lai Ping Wong, Emily Scott-Solomon, Marissa D. Fahlberg, Christina Mayerhofer, Ernst Mayerhofer, Sarah Forward, Emane Rose Assita, Giulia Schiroli, Maris Handley, Youmna Kfoury, Tsuyoshi Fukushima, Dan Li, Samuel Keyes, Azeem Sharda, Jelena Milosevic, Hiroki Kato, Pavel Ivanov, David B. Sykes, Sheldon J. J. Kwok, Ruslan I. Sadreyev, Vijay G. Sankaran, Ya-Chieh Hsu, David T. Scadden

**Affiliations:** ^1^ Center for Regenerative Medicine, Massachusetts General Hospital, 185 Cambridge Street, Boston, MA 02114, USA.; ^2^ Harvard Stem Cell Institute, 7 Divinity Avenue, Cambridge, MA 02138, USA.; ^3^ Department of Stem Cell and Regenerative Biology, Harvard University, Cambridge, MA 02138, USA.; ^4^ Department of Molecular Biology, Massachusetts General Hospital, 185 Cambridge Street, Boston, MA 02114, USA.; ^5^ Department of Genetics, Harvard Medical School, Boston, MA 02115, USA.; ^6^ LASE Innovation Inc., 335 Bear Hill Road, Waltham, MA 02451, USA.; ^7^ Center for Genomic Medicine, Massachusetts General Hospital, Boston, MA 02115, USA.; ^8^ Broad Institute of MIT and Harvard, Cambridge, MA 02142, USA.; ^9^ HSCI-CRM Flow Cytometry Core Facility, Massachusetts General Hospital, 185 Cambridge Street, Boston, MA 02114, USA.; ^10^ Division of Rheumatology, Immunology and Allergy, Brigham and Women’s Hospital, Boston, MA 02115, USA.; ^11^ Department of Medicine, Harvard Medical School, Boston, MA 02115, USA.; ^12^ Harvard Initiative for RNA Medicine, Boston, MA 02115, USA.; ^13^ Division of Hematology/Oncology, Boston Children's Hospital and Department of Pediatric Oncology, Dana-Farber Cancer Institute, Harvard Medical School, Boston, MA 02115, USA.; ^14^ Howard Hughes Medical Institute, Boston, MA 02115, USA.

## Abstract

Cell stress can increase the use of methylated guanosine (m^7^G) cap–independent, internal ribosome entry site (IRES)–mediated translation initiation relative to cap-dependent translation (IRES/Cap). Reporters that quantify IRES/Cap have demonstrated differential activity across cultured cell types and stress conditions. By generating an IRES/Cap reporter mouse, we were able to systematically evaluate IRES/Cap across distinct tissues and cell types during physiological stresses and lineage commitment. Caloric stress invoked the expected boost in IRES/Cap translation regardless of differentiation state, but unexpectedly, IRES/Cap progressively increased during hematopoietic and epithelial (hair follicle) differentiation under normal, homeostatic conditions. This was independent of total protein output or cell cycle. Even within cells of a given differentiation state, cells with lower relative IRES utilization had markedly higher multipotent capability in vivo. The RNA processing protein PTBP1 is a mediator of this translation initiation preference. Therefore, low IRES/Cap is a signature of high stemness and suggests that modulation of translation initiation participates in cell differentiation state.

## Teaser

The use of cap-independent translation initiation (relative to cap-dependent) identifies cell differentiation state in vivo.

## INTRODUCTION

Regulation of protein synthesis is a fundamental mechanism by which cells adapt to internal states and external cues (*1*). Although transcriptional control shapes long-term gene expression programs, translational control, particularly at the rate-limiting step of initiation, enables rapid and dynamic shifts in protein output (*2*). Under homeostatic conditions, most protein synthesis is driven by cap-dependent translation, which relies on the recognition of a methylated guanosine (m^7^G) cap at the 5′ end of mRNAs to recruit the eIF4F complex and initiate ribosome loading (*1*). During certain contexts, particularly cellular stress, cap-dependent translation is inhibited, and the contribution of cap-independent protein production is enhanced (*3*). One such mechanism is internal ribosome entry site (IRES)–mediated translation.

IRESes are structured sequences in the untranslated regions (UTRs) that recruit the preinitiation complex either directly or through interactions with IRES-transactivating factors (ITAFs), bypassing the need for a 5′ cap (*4*). This mode of translation initiation is best understood in the context of RNA viruses, such as encephalomyocarditis virus (EMCV). Lacking access to the nuclear capping machinery, viral RNAs can harbor IRES elements in their UTRs that co-opt host ribosomes for translation. EMCV also encodes proteases that cleave components of the host’s cap-dependent translation machinery, thereby promoting preferential translation of viral proteins via the IRES pathway (*5*–*7*).

Beyond viral systems, cap-dependent translation is known to be tightly regulated by signaling pathways, including the nutrient sensor mammalian target of rapamycin (mTOR). mTOR activity influences the availability of the cap-binding protein eIF4E by modulating the phosphorylation of the inhibitory factor 4EBP (*8*). Inhibition of mTOR activity leads to sequestration of eIF4E, suppressing cap-dependent translation while sparing cap-independent translation. Notably, a subset of mammalian mRNAs contains IRES or IRES-like elements that may be selectively translated during stress despite also having 5′ caps (*9*).

To assess the relative activity of these two modes of initiation, bicistronic reporters are the gold standard tool, consisting of a single RNA transcript with two open reading frames: The first reporter protein is translated via cap-dependent initiation, and the second reporter protein is translated via an IRES (*10*). Bicistronic reporters enable quantification of the IRES/Cap ratio (hereafter referred to as IRES/Cap), providing a readout of cap-independent IRES-mediated translation relative to cap-dependent translation. Because both fluorescent proteins are translated from the same transcript, this approach controls for mRNA abundance and promoter activity, allowing accurate comparisons of IRES/Cap under distinct conditions or cell types. However, studies comparing IRES/Cap between distinct cell types are limited to immortalized cell lines (*11*, *12*), which inadequately model normal tissue heterogeneity and physiological processes like lineage commitment. Although studies have suggested roles for IRES-mediated translation in erythroid progenitor differentiation (*13*) and in embryonic stem cells (*14*), these were restricted to in vitro systems and did not systematically assess IRES activity in the context of normal tissue differentiation. Moreover, work in lower eukaryotes has demonstrated that yeast rely on IRES-mediated translation to initiate lineage-specific programs during nutrient starvation, when cap-dependent translation is repressed (*15*), raising the possibility that cap-independent mechanisms might similarly regulate regeneration and cell fate decisions in higher-order eukaryotes.

In this study, we developed the *Translator* mouse, which uses a bicistronic reporter driven by the EMCV IRES to quantify IRES/Cap in vivo. In both hematopoietic and epithelial tissues, we found that stem cells exhibit the lowest relative IRES usage that progressively increases with progenitor lineage commitment. These findings provide a conceptual shift in how IRES-mediated translation is understood: not merely as a stress adaptation but as a homeostatically regulated process.

## RESULTS

### The *Translator* mouse quantifies IRES-mediated translation relative to canonical cap-dependent translation

**Fig. 1.**
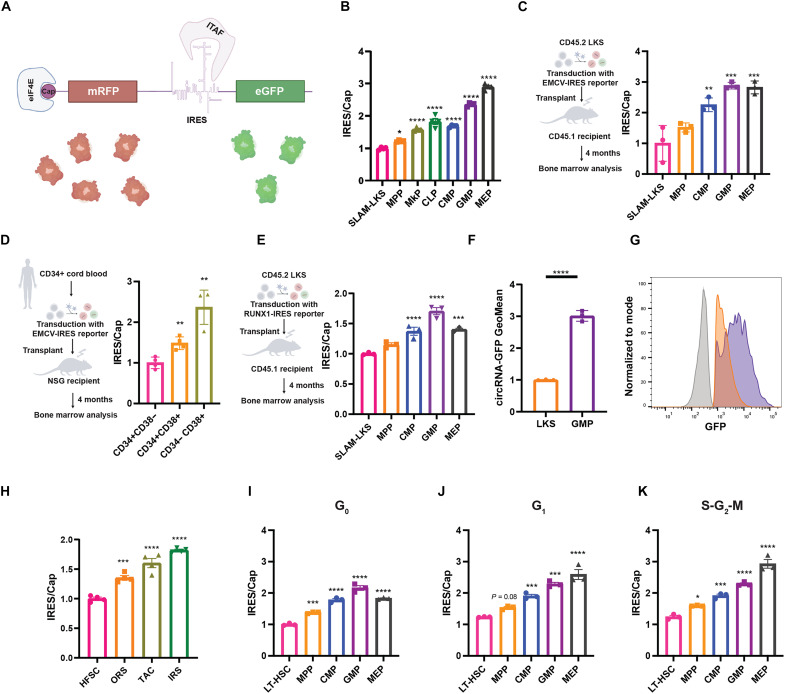
IRES/Cap increases with hematopoietic and hair follicle differentiation. (**A**) Graphical representation of the bicistronic reporter mRNA. Created in BioRender. Li, D. (2026) https://BioRender.com/pzmisd3. (**B**) EMCV-IRES/Cap in *Translator* mouse BM HSPCs. Normalized to the average of SLAM-LKS (*n* = 4). Significance versus SLAM-LKS. (**C**) EMCV-IRES/Cap of murine HSPCs derived from donor LKS transduced with lentivirus encoding bicistronic reporter and transplanted into irradiated recipients. Normalized to the average of SLAM-LKS (*n* = 3). Created in BioRender. Li, D. (2026) https://BioRender.com/d7k2abt. (**D**) EMCV-IRES/Cap in human HSPCs derived from CD34+ cord blood transduced with bicistronic reporter and transplanted into irradiated NSG recipients. Normalized to the average of CD34+CD38− (*n* = 4). Significance versus CD34+CD38−. Created in BioRender. Li, D. (2026) https://BioRender.com/d7k2abt. (**E**) RUNX1-IRES/Cap derived from donor LKS transduced with lentivirus encoding the RUNX1-bicistronic reporter and transplanted into irradiated recipients. Normalized to the average of SLAM-LKS (*n* = 3). Significance versus SLAM-LKS. Created in BioRender. Li, D. (2026) https://BioRender.com/d7k2abt. (**F** and **G**) circRNA CVB3-IRES reporter translation in cultured LKS and GMP (*n* = 3) (F) and representative histogram of GFP MFI. Gray: negative control (G). (**H**) Analysis of IRES/Cap in *Translator* mouse hair follicles, including HFSCs, ORS, TACs, and IRS. Normalized to the average of HFSC (*n* = 4). (**I** to **K**) IRES/Cap in *Translator* mouse HSPCs in G_0_ (I), G_1_ (J), and S-G_2_-M (K) and evaluated with single-cell optical barcoding (*n* = 3). Data show individual replicates and means ± SEM. Significance was assessed using a paired one-way analysis of variance (ANOVA) (B to E and H to K) and a Student’s *t* test (F). *P ≤ 0.05; **P ≤ 0.01; ***P ≤ 0.001; ****P ≤ 0.0001.

To assess the distinct usage of translation initiation in cells at various stages of differentiation, we used fluorescent bicistronic reporters that permit the simultaneous flow cytometric quantification of IRES/Cap. On a single mRNA transcript, monomeric red fluorescent protein (mRFP) is translated by cap-dependent translation and green fluorescent protein (GFP) by IRES-mediated translation (Fig. 1A). In this study, we evaluated the well-characterized EMCV-IRES, as well as the IRES within the 5′UTR of RUNX1, a transcription factor critical for hematopoiesis and hair follicle differentiation, whose IRES deletion causes embryonic lethality due to defective hematopoietic development (fig. S1, A and E) (*16*–*18*). human embryonic kidney (HEK) 293T cells transduced with lentiviral reporters confirmed simultaneous RFP and GFP fluorescence (fig. S1, B and F). To address whether the IRES had cryptic promoter activity, we removed the promoter and the mRFP coding sequence (fig. S1, C and G). The absence of GFP fluorescence in promoter-less lentiviral constructs confirmed that neither the EMCV IRES nor the RUNX1 IRES contain cryptic promoters (fig. S1, D and H). Pseudo-bulk RNA sequencing (RNA-seq) analysis confirmed transcript integrity of the EMCV reporter (table S1), consistent with previous systematic evaluation demonstrating the absence of cryptic splice sites in the EMCV and RUNX1 constructs (*19*).

To test the fidelity of these reporters, human K562 myelogenous leukemic cell lines expressing these reporters were treated with specific small molecule inhibitors of cap-dependent translation. Rocaglamide inhibits the helicase eIF4A, a critical component of the eIF4F complex, which unwinds secondary structure in the 5′UTR of mRNAs (*20*). In addition, Torin-2 inhibits mTOR and the availability of the cap-binding protein eIF4E for the cap-binding eIF4F complex formation by reducing dephosphorylation of 4EBP (*8*). As predicted, both compounds significantly increased IRES/Cap by inhibiting cap-dependent translation (fig. S1I). Therefore, although the bicistronic reporters provide relative rather than absolute protein measurements, these data indicate that their fluorescence intensities reliably capture expected shifts in protein production.

The *Translator* mouse was generated by pronuclear injection of C57BL/6 zygotes with CRISPR-Cas9 protein and guide RNA (gRNA) to introduce the mRFP-EMCV-IRES bicistronic reporter transgene into the ROSA26 locus (fig. S1J) (*21*). We focused on hematopoiesis because HSPCs have well-defined immunophenotypes and functional assays. *Translator* mouse peripheral blood counts (fig. S1, L to R), HSPC bone marrow (BM) cellularity (fig. S1S), and total BM colony-forming potential (fig. S1T) are comparable to wild-type (WT) littermates, confirming that the reporter does not perturb normal hematopoietic differentiation. As expected, Torin-2 treatment reduced cap-dependent mRFP fluorescence while sparing IRES-mediated eGFP fluorescence in immortalized guanosine monophosphates (GMPs) derived from the *Translator* mouse (fig. S1, U to W).

### IRES/Cap increases with differentiation

*Translator* mouse BM demonstrated the lowest IRES/Cap in hematopoietic stem cells (HSCs) (defined by the SLAM-LKS immunophenotype), with the ratio increasing with HSPC differentiation (Fig. 1B). In a similar manner, IRES/Cap increased with differentiation in HSPCs regenerated from murine HSCs transduced with lentivirus encoding the fluorescent bicistronic reporter and transplanted into lethally irradiated recipients (Fig. 1C). To validate this finding in human cells, we transduced CD34+ cord blood with the reporter and transplanted the transduced cells into immunodeficient mice (Fig. 1D). Consistent with murine HSPC differentiation, IRES/Cap was lowest in human primitive progenitors (CD34+CD38−) compared to differentiated progenitors (CD34−CD38+).

Next, we evaluated a mammalian IRES element from the RUNX1B gene. HSCs were transduced with lentivirus encoding the fluorescent bicistronic reporter of RUNX1B-IRES/Cap translation (Fig. 1E) and transplanted into lethally irradiated recipients. Similar to EMCV-IRES/Cap, the RUNX1B-IRES/Cap was least active in HSCs and highest in GMPs, suggesting that both viral and mammalian IRESes are differentially regulated during early hematopoietic differentiation in mice and humans.

We sought to evaluate IRES-mediated translation using a distinct reporter system. Although circular RNAs (circRNAs) are predominantly known as microRNA (miRNA) sponges and protein decoys (*22*), those being used to synthesize proteins must be translated by an IRES because they lack capped 5′ termini (*23*). Transfected circRNA encoding GFP that uses the coxsackievirus B3 (CVB3) IRES translated three times more GFP in cultured GMPs in comparison to LKS (Fig. 1, F and G). These data demonstrate that this third IRES element is also more active in lineage-committed progenitors.

After demonstrating that IRES/Cap increases with hematopoietic differentiation, we tested whether this finding existed in other tissues. The hair follicle transit amplifying cells (TACs) derived from hair follicle stem cells (HFSCs) rapidly proliferate to produce hair during adult anagen (*24*). Epidermal cells were isolated from *Translator* mice, and the ratio of IRES/Cap was compared between HFSCs, inner root sheath (IRS), TACs, and outer root sheath (ORS), defined by immunophenotype and analyzed by flow cytometry (fig. S2A). As observed in hematopoiesis, IRES/Cap was lowest in the HFSC and increased with hair follicle differentiation (Fig. 1H). IRES/Cap also increased with hair follicle differentiation when IRES/Cap was calculated using immunofluorescence imaging (fig. S2C) of HFSCs (fig. S2D) and TACs (fig. S2E). Together, these data confirm that IRES-mediated translation relative to canonical cap-dependent translation is lowest in stem cells and increases with differentiation in at least two regenerative tissues.

Adult HSCs are largely quiescent, whereas differentiated progenitors actively cycle (fig. S2E) (*25*). Because both cap-dependent and IRES-mediated translation have been shown to be associated with cell cycle status (*26*, *27*), we assessed whether differences in IRES/Cap correlated with differences in cell cycle. Flow cytometric cell cycle analysis requires fixation and permeabilization to stain with 4′,6-diamidino-2-phenylindole (DAPI) and Ki-67 (intracellular), reducing the intensity of mRFP and GFP fluorescence differently across cell types (fig. S2F). To overcome this technical limitation, we used a single-cell optical barcoding approach using semiconductor laser particles (LPs) (*28*, *29*) to measure the same cells with flow cytometry before and after fixation (fig. S2, G and H). IRES/Cap increased with HSPC differentiation, even when controlling for cell cycle status (Fig. 1, I to K), although a larger difference was observed in actively cycling cells.

### IRES/Cap is correlated with clonogenicity and regenerative potential of HSPCs

**Fig. 2.**
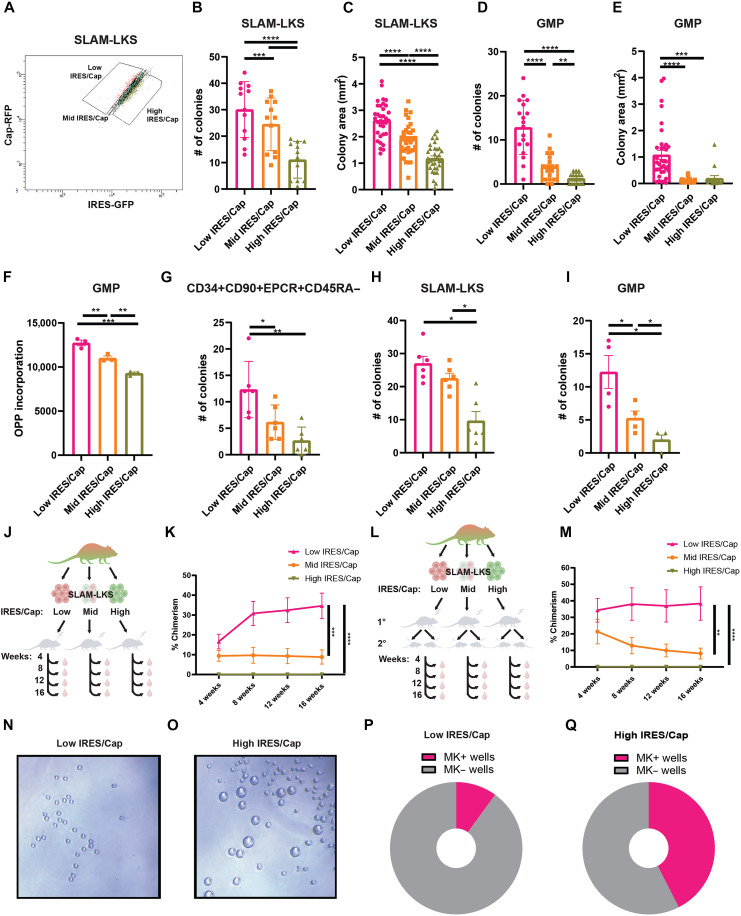
IRES/Cap is associated with clonogenicity and regenerative output of HSPCs. (**A**) Representative gating strategy for sorting low, mid, and high IRES/Cap SLAM-LKS. (**B** and **C**) Colony formation (B) and size (C, mm^2^) of *Translator* SLAM-LKS sorted on the basis of IRES/Cap (*n* = 6). (**D** and **E**) Colony formation (D) and size (E, mm^2^) of *Translator* GMP sorted on the basis of IRES/Cap (*n* = 9). (**F**) Total protein synthesis rates of *Translator* GMP based on IRES/Cap measured by AF647-OPP MFI (*n* = 3). (**G**) Colony formation of EMCV-IRES reporter-transduced CD34+CD90+EPCR+CD45RA− human cord blood sorted on the basis of IRES/Cap (*n* = 3). (**H** and **I**) Colony formation of SLAM-LKS (H) and GMPs (I) based on RUNX1-IRES/Cap sorted from transplant recipients (*n* = 3 and 2, respectively). (**J**) Primary competitive transplant schema. Twenty CD45.2 SLAM-LKS sorted on the basis of IRES/Cap versus 200,000 CD45.1 whole BM cells in lethally irradiated CD45.1 recipients. Peripheral blood chimerism analyzed every four weeks through week 16. Created in BioRender. Li, D. (2026) https://BioRender.com/d7k2abt. (**K**) Total chimerism *Translator* SLAM-LKS based on IRES/Cap in primary recipients evaluated through week 16 (*n* = 12). (**L**) Secondary competitive transplant schema. One million total BM cells were collected from primary recipients (16 weeks posttransplantation) and transplanted to lethally irradiated CD45.1 recipients. Created in BioRender. Li, D. (2026) https://BioRender.com/d7k2abt. (**M**) Total chimerism *Translator* SLAM-LKS based on IRES/Cap in secondary recipients through week 16 (*n* = 12). (**N** and **O**) Representative image of the culture of a single low (N) or high (O) IRES/Cap SLAM-LKS after 5 days of culture. (**P** and **Q**) Pie charts depicting the percentage of wells with megakaryocytes (MK) after 5 days of culturing a single low (P) or high (Q) IRES/Cap *Translator* SLAM-LKS (*n* = 20). Data show individual replicates and means ± SEM. *P ≤ 0.05; **P ≤ 0.01; ***P ≤ 0.001; ****P ≤ 0.0001. Significance was assessed using a two-way ANOVA with Dunnett’s one-way ANOVA (B to I) or an ANOVA with Tukey’s multiple comparisons test (K and M).

To assess whether IRES/Cap is associated with hematopoietic function, we performed soft agar colony assays, plating HSPCs with the 20% lowest and 20% highest ratio of IRES/Cap compared to the average (“mid”) (Fig. 2A). *Translator* mouse SLAM-LKS with low IRES/Cap produced significantly more colonies compared to those with the high IRES/Cap in both primary (Fig. 2B) and secondary (fig. S3A) colony assays. Low IRES/Cap SLAM-LKS colonies were also significantly larger than high IRES/Cap SLAM-LKS (Fig. 2C and fig. S3B). *Translator* mouse GMPs with low IRES/Cap also produced significantly more colonies (Fig. 2D) that were greater in size (Fig. 2E) compared to high IRES/Cap GMPs. Next, we evaluated total rates of protein synthesis in GMPs sorted on the basis of IRES/Cap. GMPs with low IRES/Cap had significantly greater rates of total protein synthesis, as measured by *O*-propargyl-puromycin (OPP) incorporation (Fig. 2F). Human cultured HSPCs with low IRES/Cap produced significantly more colonies than those with high IRES/Cap (Fig. 2G). Last, to evaluate whether this trend was specific to the EMCV-IRES, we sorted HSPCs on the basis of RUNX1-IRES/Cap; SLAM-LKS and GMPs with lower utilization of the RUNX1-IRES relative to cap-dependent translation produced more colonies (Fig. 2, H and I). Collectively, low IRES/Cap HSPCs have an increased proliferative and regenerative capacity ex vivo.

To evaluate the impact of IRES/Cap on HSC function in vivo, we transplanted SLAM-LKS on the basis of IRES/Cap in competition with CD45.1 marrow into lethally irradiated recipients (Fig. 2J). Low IRES/Cap SLAM-LKS resulted in significantly greater peripheral blood chimerism over 16 weeks across all lineages compared to average (mid) IRES/Cap and high IRES/Cap (Fig. 2K and fig. S3, C to E). The self-renewal of LT-HSC defined by SLAM-LKS based on IRES/Cap was evaluated by performing serial transplantation (Fig. 2L). Low IRES/Cap SLAM-LKS had persistent chimerism in secondary recipients, whereas mid IRES/Cap had reduced regenerative potential after 16 weeks (Fig. 2M and fig. S3, F to H). The relative decline in myeloid chimerism observed at later time points is consistent with similar reconstitution kinetics in fixed-dose competitive transplantation assays (*30*–*32*).

To investigate a potential reason behind the low chimerism observed from high IRES/Cap SLAM-LKS, we sorted SLAM-LKS on the basis of IRES/Cap (fig. S3I) and grew them in HSC expansion liquid cultures. After 5 days of culture, high IRES/Cap SLAM-LKS produced significantly more megakaryocytes than low IRES/Cap (Fig. 2, N to Q).

Collectively, these data indicate that IRES/Cap distinguishes functional subsets of HSC (SLAM-LKS). Low IRES/Cap SLAM-LKS have the greatest capacity for long-term reconstitution of blood in serial transplantations and high IRES/Cap SLAM-LKS are megakaryocyte primed. Therefore, the relative use of these translation initiation mechanisms is indicative of functional distinctions between immunophenotypically identical cells.

### IRES utilization is associated with the expression of differentiation-related genes

**Fig. 3.**
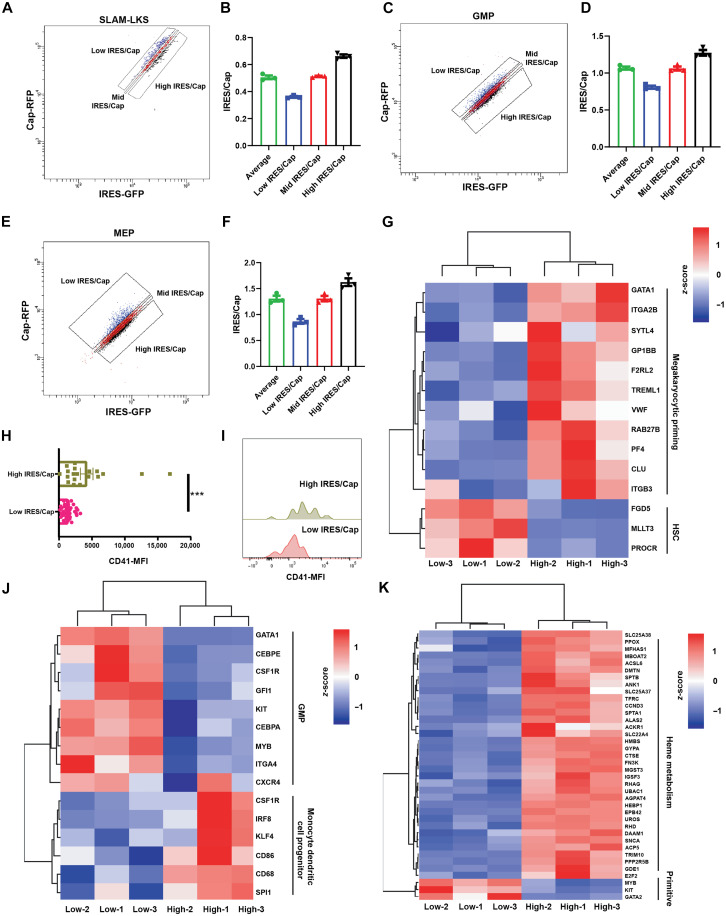
IRES/Cap is associated with differentiation gene expression. (**A**) Representative gating strategy for sorting SLAM-LKS based on IRES/Cap by flow cytometry. (**B**) Quantification of IRES/Cap based on gating strategy in (A). IRES/Cap is normalized to that of the entire population of SLAM-LKS (*n* = 3). Data show means ± SEM. (**C**) Representative gating strategy for sorting GMP based on IRES/Cap by flow cytometry. (**D**) Quantification of IRES/Cap based on gating strategy in (D). IRES/Cap is normalized to that of the entire population of GMP (*n* = 3). Data show means ± SEM. (**E**) Representative gating strategy for sorting MEP based on IRES/Cap by flow cytometry. (**F**) Quantification of IRES/Cap based on gating strategy in (G). IRES/Cap is normalized to that of the entire population of MEP (*n* = 3). Data show means ± SEM. (**G**) DEGs enriched in high IRES/Cap SLAM-LKS compared to low IRES/Cap SLAM-LKS (*n* = 3). (**H**) CD41-MFI on index-sorted low and high IRES/Cap *Translator* SLAM-LKS. (**I**) Representative histogram of CD41-BV711 on index-sorted low and high IRES/Cap *Translator* SLAM-LKS measured by flow cytometry. (**J**) DEGs enriched in high IRES/Cap GMP compared to low IRES/Cap GMP (*n* = 3). (**K**) DEGs enriched in high IRES/Cap MEP compared to low IRES/Cap MEP (*n* = 3). Data show individual replicates and means ± SEM. Significance was assessed using a Student’s *t* test (H). ***P ≤ 0.001.

Because IRES/Cap correlates with differentiation status within a particular immunophenotype, we evaluated the transcriptome of HSPCs sorted on the basis of their relative use of IRES-mediated translation. Translator SLAM-LKS, GMP, and megakaryocyte-erythroid progenitor (MEP) gated on the basis of the ratio of IRES/Cap were evaluated by single-cell transcriptomic analysis (100 cells per condition; Fig. 3, A to F, and table S2). Genes associated with megakaryocyte lineage priming were up-regulated in high IRES/Cap SLAM-LKS (*33*, *34*), compared to low IRES/Cap SLAM-LKS, which had greater expression of genes associated with stemness, including Fdg5, Mllt3, and Procr (Fig. 3G) (*35*–*37*). CD41 protein, a cell surface marker of megakaryocyte priming (*38*), was quantified in SLAM-LKS. High IRES/Cap SLAM-LKS had significantly greater CD41 protein compared to those with low IRES/Cap (Fig. 3, H and I), which is consistent with the lower chimerism of high IRES/Cap SLAM-LKS in competitive transplant experiments (Fig. 2K) and high megakaryocytic output ex vivo (Fig. 2, N to Q). Last, low IRES/Cap SLAM-LKS had significantly greater expression of genes included in Hallmark 2020 terms associated with inflammation, specifically interferon-γ signaling, which is known to be expressed in HSCs (fig. S4, A, B, and G to K) (*39*).

Differentially expressed genes (DEGs) enriched in high IRES/Cap GMPs were functionally associated with monocyte dendritic cell progenitors (Csf1R, Irf8, Flg4, CD86, CD68, and Spi1) (Fig. 3J and fig. S4D), consistent with myeloid differentiation (*40*–*42*). Conversely, low IRES/Cap GMPs had higher expression of genes critical for the myeloid progenitor state (Gata1, Cebpe, Csf1r, Gfi1, Kit, Cebpa, Myb, Itga4, and Cxcr4) (Fig. 3J and fig. S4C). Similarly, high IRES/Cap MEPs had significantly greater expression of genes involved in heme metabolism compared to low IRES/Cap, suggesting that IRES/Cap is positively correlated with MEP differentiation (Fig. 3K and fig. S4, E and F). These transcriptomic analyses collectively support our findings that HSPCs with low relative IRES utilization are more primitive and poised for high cell output, whereas those with high relative IRES utilization are more differentiated and perform effector functions.

### Relative IRES usage is positively regulated by PTBP1

**Fig. 4.**
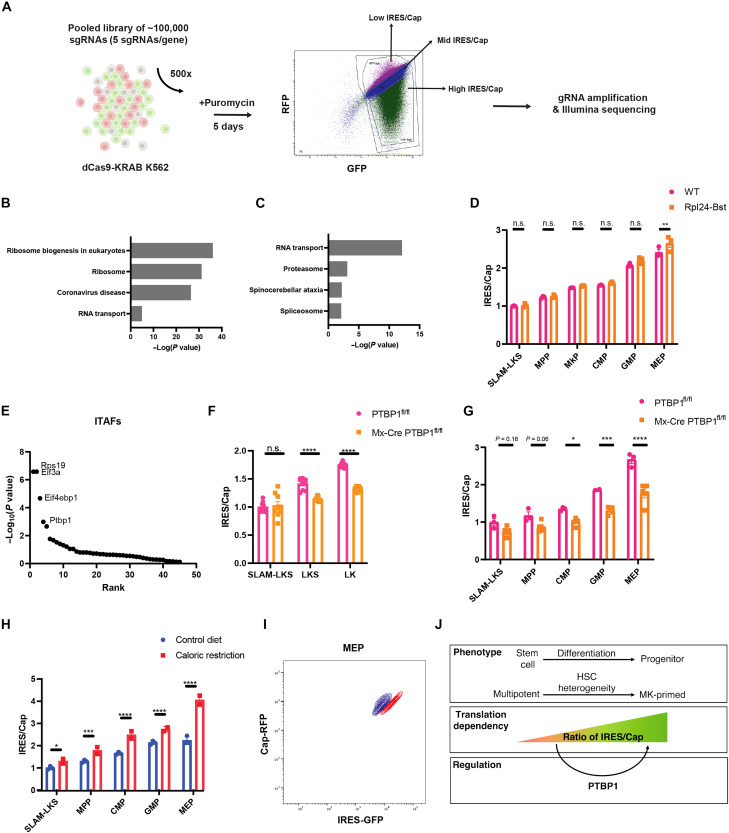
IRES/Cap is regulated by PTBP1 and caloric restriction. (**A**) Graphical representation of a genome-wide CRISPRi screen workflow to identify positive and negative regulators of IRES/Cap. Created in BioRender. Li, D. (2026) https://BioRender.com/d7k2abt. (**B**) Top KEGG terms associated with genes targeted by sgRNAs enriched in the “low IRES/Cap” gate (i.e., putative positive regulators of IRES/Cap) (*n* = 3). (**C**) Top KEGG terms associated with genes targeted by sgRNAs enriched in the “high IRES/Cap” gate (i.e., negative regulators of IRES/Cap) (*n* = 3). (**D**) Analysis of EMCV-IRES/Cap in *Translator* Rpl24-Bst HSPCs compared to WT littermate controls (*n* = 3). (**E**) Known ITAFs (*4*) ranked in order of significance as putative positive regulators of IRES/Cap in K562 (*n* = 3). (**F**) EMCV-IRES/Cap in PVA-expanded PTBP1^fl/fl^ and Mx-Cre PTBP1^fl/fl^ HSPCs measured by flow cytometry (*n* = 4). (**G**) EMCV-IRES/Cap in PTBP1^fl/fl^ and Mx-Cre PTBP1^fl/fl^ HSPCs regenerated from transduced LKS in CD45.1 recipients (*n* = 3 for PTBP1^fl/fl^ and *n* = 5 for Mx-Cre PTBP1^fl/fl^). (**H**) EMCV-IRES/Cap in *Translator* mouse HSPCs for animals fed with control diet (*n* = 3) or those that underwent caloric restriction (*n* = 2). Normalized to the average of SLAM-LKS. Significance versus SLAM-LKS from control diet. (**I**) Representative flow plot of MEPs analyzed from mice that were fed normal diet or underwent caloric restriction. (**J**) Illustration depicting the key findings that IRES utilization relative to cap-dependent translation (IRES/Cap) increases with differentiation, which is partially regulated by PTBP1. Data show individual replicates and means ± SEM. Significance was assessed using a two-way ANOVA (D and F to H). *P ≤ 0.05; **P ≤ 0.01; ***P ≤ 0.001; ****P ≤ 0.0001; n.s., not significant.

ITAFs are capable of regulating IRES-mediated protein production (*4*). To identify specific regulators of IRES/Cap in hematopoietic cells, we performed a genome-wide CRISPR interference (CRISPRi; dCas9-KRAB) screen on K562 cells expressing the EMCV-IRES bicistronic reporter (Fig. 4A and table S3). The dCas9 activity was validated using guides to known targets (fig. S4L). Following transduction with a pooled gRNA library, K562 cells were sorted on day 5 based on the ratio of IRES/Cap, and gRNAs were sequenced. Putative positive regulators of IRES/Cap [i.e., Kyoto Encyclopedia of Genes and Genomes (KEGG) terms associated with gRNA enriched in low IRES/Cap] were ribosome biogenesis regulators (Fig. 4B), whereas putative negative regulators were enriched for RNA transport proteins (Fig. 4C). These findings implicated ribosome abundance as a key regulator of differential IRES translation in HSPCs.

Stem cells produce less total protein than more differentiated progenitors (*43*–*46*). On the basis of the CRISPRi screen results, we questioned whether IRES/Cap reflects differences in total rates of protein synthesis, rather than differentiation status. To address this question, we crossed the *Translator* mouse to the Rpl24^Bst/+^ model, which has well-characterized defects in ribosome biogenesis, slower rates of total protein synthesis in HSPCs, and impaired HSC function (*43*). IRES/Cap was comparable between WT and Rpl24^Bst/+^ HSPCs, suggesting that perturbation of total translation rates by interfering with ribosome function does not alter IRES/Cap (Fig. 4D).

We evaluated how known ITAFs influenced the ratio of IRES/Cap (Fig. 4E) (*4*). Ranking fourth, PTBP1 inhibition reduced IRES/Cap. PTBP1 is a known regulator of ribosomal biogenesis and a stabilizer of IRES elements containing the CUUU motif (*47*), which is present in both the EMCV and RUNX1 IRESes. Blood-specific deletion of PTBP1^fl/fl^ by Mx-Cre impairs red blood cell (RBC) production and HSC function in serial transplantations (*48*), suggesting that PTBP1 functionally regulates hematopoietic differentiation.

We cultured and transplanted Mx-Cre PTBP1^fl/fl^ and PTBP1^fl/fl^ HSCs transduced with the EMCV-IRES reporter and evaluated the ratio of IRES/Cap after 5 days of liquid culture and 4 months posttransplantation. IRES/Cap is lower in more differentiated Mx-Cre PTBP1^fl/fl^ HSPCs in vitro (Fig. 4F) and in vivo (Fig. 4G) compared to WT PTBP1^fl/fl^. This finding was replicated from Translator-iCAS9 HSPCs transduced with PTBP1-targeting or nontargeting guides and transplanted into lethally irradiated recipients (fig. S4, M and N). Therefore, PTBP1 is one of the multiple critical components of the IRES/Cap regulatory mechanisms in hematopoiesis.

### Caloric restriction increases IRES/Cap in both stem and progenitor cells

IRES translation persists when mTOR signaling is pharmacologically inhibited or reduced under conditions of nutrient deprivation (*49*). In line with this observation, IRES/Cap increased in each subset of hematopoietic cells in the setting of calorie restriction (Fig. 4, H and I) or short-term fasting (fig. S4O). These data parallel the effect of chemical inhibitors of cap-dependent translation in K562 cells (fig. S1I) and suggest that, although IRES utilization is developmentally constrained in hematopoiesis, it can be activated under stress. Calorie restricted animals also had significant reductions in RBC and white blood cell (WBC) counts, although the platelet count was preserved (fig. S4, P to R). Ultimately, we propose that IRES utilization is enhanced not only during stress but also as a critical signature of normal differentiation and homeostasis.

## DISCUSSION

The *Translator* mouse revealed that HSCs had the lowest IRES/Cap ratios and that this ratio increased with differentiation. Manipulation of total translation rates, such as through the Rpl24^Bst/+^ genotype, was insufficient to alter IRES/Cap ratios across hematopoiesis. Collectively, these data indicate that cap-dependent translation machinery and ITAFs, like PTBP1, are regulated distinctly in differentiating tissues, influencing relative IRES utilization independently of the overall translation rates (Fig. 4J).

Because IRES-mediated translation is differentially used throughout hematopoietic and hair follicle differentiation, it is likely that this mechanism plays a broader role in other stem cell–dependent contexts. Furthermore, the relative use of IRES correlates with functional heterogeneity within immunophenotypically identical cells. IRES/Cap measured by the *Translator* mouse bicistronic reporter identifies a subset of SLAM-LKS capable of long-term regenerative output. Notably, higher IRES/Cap among immunophenotypic HSCs associates with accelerated differentiation and a complete failure to function as stem cells. Therefore, IRES use negatively correlates with stemness and may serve as a capacitor for differentiation.

In murine hematopoietic differentiation, translation regulation is critical to the activation of stemlike megakaryocytic progenitors in response to acute inflammation (*34*), and RUNX1 has been shown to be a critical regulator of megakaryocyte-primed HSC differentiation (*50*). Because RUNX1B protein expression is regulated in part by an IRES, these data may explain why high IRES/Cap SLAM-LKS produced significantly more megakaryocytes ex vivo and very low chimerism in primary and secondary transplant recipients. In addition, HSPCs with greater expression of RUNX1B protein have lower clonogenicity (*51*), as we observed with HSPCs with greater RUNX1B-IRES activity. Regulators of erythroid differentiation Bag1 and Csde1 contain putative IRES elements in their 5′UTRs that are regulated by Rps19, an ITAF that scored significantly as a positive regulator of IRES/Cap in K562 (*13*). Collectively, these data suggest that more differentiated progenitors, particularly along the megakaryocytic and erythroid lineages, use IRES-mediated protein production to enable differentiation and cell production. However, quantification of endogenous IRES-mediated protein production is technically infeasible with methods such as ribosome profiling, given that all mRNAs are capped (including those with IRESes) and the coding sequence of IRES-containing isoforms is highly homologous with non-IRES–containing isoforms.

Although our CRISPRi screen identified multiple ITAFs that regulate IRES/Cap ratios, we focused our functional validation studies on PTBP1 due to several key advantages: its well-characterized role in stabilizing IRES elements containing the CUUU motif present in both our EMCV and RUNX1 constructs, the availability of viable knockout mice with a characterized hematopoietic phenotype (*48*), and the fact that its role in IRES regulation specifically during differentiation has not been well explored. It is highly likely that additional ITAFs beyond PTBP1 contribute to the increase in IRES/Cap with differentiation because PTBP1^−/−^ differentiated progenitors do not have equivalent IRES/Cap to WT HSCs. Our CRISPRi screen in K562 cells identified several additional positive regulators of IRES/Cap, including RIOK2 and UTP18. RIOK2 is particularly intriguing given that its knockout causes anemia defects in mouse and human (*52*, *53*). In addition, its expression increases with hematopoietic differentiation and is highest in MEPs, consistent with the elevated IRES/Cap ratios observed in more differentiated populations. Although RIOK2 has not been previously linked to IRES regulation, its role as a master transcription factor coordinating erythroid differentiation suggests that it may simultaneously regulate both transcriptional and translational programs. UTP18 also increases in expression during differentiation and has been shown to up-regulate IRES translation under stress conditions by shuttling to the cytoplasm and promoting translation of IRES-containing transcripts (*54*). In addition, it is possible that proteins negatively regulate the IRES/Cap across differentiation; our screen identified EIF3, which has known impacts on cap-dependent translation (*55*). Future studies could parse the contribution to ITAFs beyond PTBP1 during differentiation.

It is important to note that the translational initiation is more complex than a simple binary switch between canonical cap-dependent and IRES-mediated translation. A recent work has identified alternative cap-dependent translation mechanisms, including DAP5/eIF3d-mediated translation that functions independently of eIF4E and mTORC1 (*56*). Under conditions of mTORC1 inhibition or cellular stress, when eIF4E is sequestered by 4E-BPs, certain mRNAs can still undergo cap-dependent translation through recruitment of the DAP5/eIF3d complex. DAP5 has also been linked to the translation of IRESes (*57*). Future studies dissecting the relative contributions of these distinct cap-dependent mechanisms could provide additional insights into the translational regulatory networks governing differentiation.

PTBP1 has been associated with enhancing virus production, including integrating viruses like HIV (*58*) and nonintegrating viruses like dengue virus (*59*), which both have reported IRES elements (*60*–*63*). Therefore, low stem cell expression of PTBP1 may protect them from producing viral proteins that depend on IRES translation. Given their self-renewing capabilities, stem cells may constrain the translation of IRES-mediated viral proteins to protect themselves from durable genomic modifications that pose oncogenic risk. HSC infection by nonintegrating RNA viruses, like dengue virus, has been shown to impair the regenerative capacity of transplanted HSCs lacking intact interferon signaling (*39*). Because stem cells have low tropism to many viruses as a mechanism of protection, it is challenging to evaluate whether lower viral IRES-mediated translation is an additional layer of protection. However, it is notable that human HSCs have been shown to be resistant to HIV infection despite documented receptor and co-receptor expression (*15*, *64*).

Furthermore, expression of endogenous retroviral genetic elements has recently been demonstrated to be important in HSC response to stress (*65*). The expression of those likely triggers an interferon response that may be adaptive, but translation of retroviral proteins to enable further integration could be problematic for HSC genomic stability. Notably, low IRES/Cap SLAM-LKS have significantly greater expression of interferon pathway genes associated with viral protection compared to high IRES/Cap SLAM-LKS (fig. S4, G to K). Therefore, it is intriguing to consider whether the restriction on IRES-mediated translation in stem cells serves as a means of preserving stem cell genetic integrity.

## MATERIALS AND METHODS

### Mice

All animal experiments were approved by the Institutional Animal Care and Use Committee (IACUC) at Massachusetts General Hospital (IACUC protocol #2016N000085). Mice were housed in a temperature-and humidity-controlled environment with a 12-hour light/12-hour dark cycle and food and water ad libitum. WT CD45.2 C57BL/6J (strain #000664), CD45.1 (B6.SJL-*Ptprc^a^ Pepc^b^*/BoyJ; strain #002014), KH2/iCas9 [B6;129S4-Gt(ROSA)26Sor^tm1(rtTA*M2)Jae^ Col1a1^tm1(tetO-cas9)Sho^/J; strain #029415], Rpl24^Bst^ (C57BLKS-Rpl24^Bst^/J; strain #000516), and NSG mice (NOD.Cg-PrkdcscidIl2rgtm1Wjl/SzJ; strain #005557) were purchased from the Jackson Laboratory. Mice were gender matched and 8 to 14 weeks of age in all experiments unless stated otherwise.

### Generation of the *Translator* mouse

The *Translator* mouse was generated as previously described (*21*) by the Harvard Genome Modification Facility. The knock-in construct was modified from pR26CAG/GFP Dest (#74286, Addgene) by VectorBuilder to include the bicistronic fluorescent reporter (mRFP-EMCV IRES-eGFP) downstream of a CAG promoter followed by a puromycin transcription stop cassette flanked by Loxp sites. The puromycin transcription stop cassette was removed by an in vitro Cre Recombinase reaction (#M0298, New England BioLabs), per the manufacturer’s instructions. The donor DNA consists of a 1083–base pair (bp) left homology arm and a 4341-bp right homology arm to target to the ROSA26 locus. Pronuclear injection of the donor DNA paired with a purified Cas9 Nuclease V3 protein (#1081058, Integrated DNA Technologies) and a gRNA targeting ROSA26 (ACUCCAGUCUUUCUAGAAGA) was performed on C57BL/6J zygotes. The transgenic progenies were evaluated for cassette integration by flow cytometry on peripheral blood by retro-orbital bleeding using heparinized microhematocrit capillary tubes (#22-362-566, Fisherbrand) and depleted of RBCs by ACK lysis (#18-156-721, Quality Biological). Heterozygous transgene positive founders were bred with WT CD45.2 C57BL/6J mice purchased from the Jackson Laboratory. An immortalized GMP cell line was generated using the ER-HOXB8 system, as previously described (*66*).

### Flow cytometry analysis and sorting

Mice were euthanized by CO_2_ asphyxia. For hematopoietic studies, total BM was collected by crushing the tibias, femurs, hips, humeri, and spine. Lineage positive cells were depleted with a lineage cell depletion kit (#130-090-858, Miltenyi Biotec), as per the manufacturer’s instructions. Lineage-depleted marrow was stained in phosphate-buffered saline (PBS) supplemented with 2% fetal bovine serum (FBS) using the following antibodies: Sca1-BV785 (Clone D7, #108139, BioLegend, 1:200), cKit-APC-eFluor 780 (Clone 2B8, #47-1171-82, Invitrogen, 1:100), CD150-PE-Cy7 (Clone TC15-12F12.2, #115914, BioLegend, 1:200), CD48-BUV737 (Clone HM48-1, #749666, BD, 1:200), CD16/32-BV605 (Clone 2.4G2, #563006, BD, 1:200), CD34-AF647 (Clone RAM34, #560230, BD, 1:30), CD41-BV711 (Clone MWReg30, #740712, BD, 1:200), CD127− eFluor 450 (Clone A7R34, #48-1271-82, eBioscience, 1:100), and biotinylated lineage cocktail composed of equal parts CD8a (Clone 53-6.7, #553029, BD), CD3ε (Clone 145-2C11, #553060, BD), CD45R (Clone RA3-6B2, #553086, BD), GR1 (Clone RB6-8C5, #553125, BD), CD11b (Clone M1/70, #553309, BD), Ter119 (Clone Ter-119, #553672, BD), and CD4 (Clone GK1.5, #553728, BD) (1:50), followed by the Streptavidin-AF700 conjugate (#S21383, Invitrogen, 1:300). Dead cells were excluded from analysis by staining with DAPI (#62248, Thermo Fisher Scientific, 0.1 μg/ml). The following cell surface markers were used to define: SLAM-LKS (DAPI−Lin−Sca1+cKit+CD150+CD48−), MPP (DAPI−Lin−Sca1+cKit+CD150−CD48+), LKS (DAPI−Lin−Sca1+cKit+), MkP (DAPI−Lin−Sca1−cKit+CD150+CD41+), CMP (DAPI−Lin−Sca1− cKit+CD34+CD16/32−), CLP (DAPI Lin Sca1medcKitmedCD127+), GMP (DAPI−Lin−Sca1−cKit+CD34+CD16/32+), and MEP (DAPI−Lin−Sca1−cKit+CD34−CD16/32−). Marrow was stained for 1.5 hours for primary antibodies and 15 min for Streptavidin secondary stains at 4°C. For analysis and sorting of human cord blood transplantations into NSG mice, marrow was depleted of RBCs by performing ACK lysis (#18-156-721, Quality Biological) and stained in PBS supplemented with 2% FBS using the following antibodies: hCD19-BV711 (Clone HIB19, #302246, BioLegend, 1:100), hCD3-BV711 (Clone UCHT1, #300463, BioLegend, 1:100), hCD34-APC-Cy7 (Clone 581, #343514, BioLegend, 1:100), hCD38 PE-Cy7 (Clone HIT2, #303516, BioLegend, 1:100), hCD90-BV421 (Clone 5E10, #328122, BioLegend, 1:50), and hCD45RA-BUV737 (Clone HI100, #564442, BD, 1:100). For analysis and sorting of human cord blood for colony assays, cultured and transduced CD34+ cells were stained with hCD34-APC-Cy7 (Clone 581, #343514, BioLegend, 1:100), hCD45RA-BUV737 (Clone HI100, #564442, BD, 1:100), hCD90-BV711 (Clone 5E10, #328139, BioLegend, 1:50), and hCD201-APC (Clone RCR-401, #351906, BioLegend, 1:25). Dead cells were excluded from analysis by staining with DAPI (#62248, Thermo Fisher Scientific, 0.1 μg/ml).

For hair follicle analysis, translator mice in the second anagen (P29-P30) were euthanized by CO_2_ asphyxia. Mouse back skin was dissected, and the fat layer was scraped using a surgical scalpel (#29550, Exel Int). The skin was incubated on an orbital shaker (75 rpm) dermal side down in Collagenase (0.25%, Sigma-Aldrich) diluted in Hanks’ balanced salt solution for 1 hour at 37°C. Dermal cells were obtained by scraping the dermal side with a dulled surgical scalpel. Dermal cells were then spun at 500*g* at 37°C for 5 min and resuspended in 0.25% Trypsin-EDTA (#25200072, Thermo Fisher Scientific) and incubated at 37°C for 30 min with agitation. Dermal single-cell suspensions were obtained by filtering trypsin suspension through 70- and 40-μm filters. For epidermal cells, the post–Collagenase digested skin was incubated on an orbital shaker (75 rpm) dermal side down in 0.25% Trypsin-EDTA (#25200072, Thermo Fisher Scientific) and incubated at 37°C for 30 min. Epidermal single-cell suspensions were obtained by scraping the epidermal side with a dulled surgical scalpel and filtering through 70- and 40-μm filters. Dermal and epidermal cells were combined, blocked with CD16/32 Fc (#553142, BD) and incubated for 15 min at 4°C. Cells were incubated with anti-CD140a-Biotin (eBioscience, 13-1401-82, 1:200) for 30 min at 4°C. CD140a+ cells were depleted using Dynabeads M-280 Streptavidin (#11205D, Invitrogen). Cells were incubated with CD49f-APC-Integrin α6 (#313616 BioLegend, 1:500), CD45.2-APC-Cy7 (#109824 BioLegend, 1:250), Sca1-BV785 (#108139 BioLegend, 1:200), and CD34-BV421 (#562608 BD, 1:50). Dead cells were excluded from analysis by staining with DAPI (#62248, Thermo Fisher Scientific, 0.1 μg/ml). The following cell surface markers were used to define: HFSC (DAPI−CD45.2−CD49f+CD34+Sca1−), ORS (DAPI−CD45.2−CD49fhiSca1−), TACs (DAPI−CD45.2−CD49f^mid^CD34−Sca1−), and IRS (DAPI−CD45.2− CD49f^lo^CD34−Sca1−). All cytometry analysis above was performed using FlowJo software version 10.9.0.

When evaluating the ratio of IRES/Cap, the geometric mean fluorescence intensity (geo MFI) was calculated on reporter positive cells for fluorescein isothiocyanate (FITC) (GFP) and phycoerythrin (PE)–Texas Red (mRFP1) using FlowJo and then divided. When sorting HSPCs based on IRES/Cap, the ratio of was calculated for the entire population of interest. Then, gates were drawn to include ~20% of cells with the lowest IRES/Cap, 20% with the median IRES/Cap (equal to IRES/Cap of the parent population), and 20% with the highest IRES/Cap.

### Cell cycle analysis

BM was lineage depleted, and HSPCs were immunophenotyped as described above. Lineage-depleted marrow was stained with Sca1-PE-Cy5 (Clone D7, #108109 BioLegend, 1:200), cKit-BV650 (Clone ACK2, #752697, BD Biosciences, 1:100), CD150-PE-Cy7 (Clone TC15-12F12.2, #115914, BioLegend, 1:200), CD48-PerCP-Cy5.5 (Clone HM48-1, #103421, BioLegend, 1:200), CD16/32-BV605 (Clone 2.4G2, #563006, BD Biosciences, 1:200), CD34-AF647 (Clone RAM34, #560230, BD, 1:30), and the aforementioned lineage cocktail, followed by the Streptavidin-AF700 conjugate (#S21383, Invitrogen, 1:300). Before fixation and permeabilization, live marrow samples were barcoded with biotin-coated LPs as described previously (*29*) via biotinylated CD45 (Clone 30-F11, #103104, BioLegend), CD105 (Clone MF7/18, #13-1051-85, eBioscience), CD150 (Clone TC15-12F12.2, #115908, BioLegend), CD41 (Clone MWReg30, #133930, BioLegend), and H-2Kb/H2-Db (Clone 5041.16.1, #PIMA517998, Invitrogen) and purified streptavidin (#405151, BioLegend). Afterward, samples were stained for viability, acquired, and captured on a multipass flow cytometer (LASE Innovation Inc.). The barcoded BM samples were then fixed and permeabilized using the BD Cytofix/Cytoperm Fixation/Permeabilization Kit as described above and stained for AF555-Ki-67 (Clone B56, #558617, BD Biosciences, 1:10) and DAPI. In the second cycle’s measurement, three biological replicates were acquired at 10 μl/min to maximize DAPI resolution.

Single-color compensation controls were acquired the same day with UltraComp eBeads, ArC beads, and GFP BrightComp eBeads (Invitrogen) at gain settings dictated by instrument voltration. A total of ~7500 events per compensation control sample were acquired to generate the compensation matrix used for each cycle. For each sample analyzed with the multipass method, the FCS files from both cyclic measurements (pre- and postfix/perm) were concatenated by LP barcode by the LP matching algorithm as described previously (*29*). The full compensation matrix was manually constructed by concatenating two copies of the original 12 × 12 compensation matrix into a 24 × 24 element matrix where elements corresponding to fluorophores of markers measured in different cycles were assigned to 0. When sorting HSPCs based on IRES/Cap, the ratio was calculated for the entire population of interest. The positive Ki-67 gate was distinguished by the Alexa Fluor 555 isotype control (#9641, Cell Signaling Technologies, 1:20), which was stained using the same antibodies as the samples, except using anti-IgG1 Alexa Fluor 555 in place of Ki-67 Alexa Fluor 555.

### Culture of murine LKS and GMP

GMPs were sorted as described from WT (C57BL/6J) and cultured in StemSpanSFEMII (Stem Cell Technologies) supplemented with penicillin (#15140-122, Gibco, 100 IU/ml), streptomycin (#15140-122, Gibco, 100 IU/ml), and glutamine (#25030081, Gibco, 2 mM) in addition to mouse recombinant cytokines: SCF (#250-03, PeproTech, 100 ng/ml), IL-3 (#213-13, PeproTech, 20 ng/ml), and IL-6 (#216-16, PeproTech, 20 ng/ml). LKS were sorted as described above and cultured in StemSpanSFEMII (Stem Cell Technologies) supplemented with penicillin (#15140-122, Gibco, 100 IU/ml), streptomycin (#15140-122, Gibco, 100 IU/ml), and glutamine (#25030081, Gibco, 2 mM) in addition to mouse recombinant cytokines: SCF (#250-03, PeproTech, 100 ng/ml), IL-3 (#213-13, PeproTech, 20 ng/ml), TPO (#315-14, PeproTech, 50 ng/ml), and FLT3 ligand (#250-31L, PeproTech, 100 ng/ml).

### In vitro protein translation assay

GMPs sorted on the basis of the ratio of IRES/Cap from lineage-depleted *Translator* mouse BM were incubated in a humidified 37°C incubator for 30 min in media containing 20 μM OPP (MedChemExpress). Cells were stained with the LIVE/DEAD Fixable Blue stain (Thermo Fisher Scientific) according to the manufacturer’s protocol followed by fixation using the Fixation/Permeabilization kit (BD Biosciences). After fixation, cells were washed and permeabilized using 1X perm/wash buffer (BD Biosciences). Cells were stained for OPP using the Click-iT Plus Alexa Fluor 647 Picolyl azide kit (Invitrogen) according to the manufacturer’s protocol and using 40% copper(II) sulfate and analyzed using a BD FACSAria II.

### CircRNA translation assay

Equal numbers of C57BL/6J LKS and GMPs were sorted from lineage-depleted BM and cultured as described above. One microgram of EGFP-circRNA (Creative Biogene, PMCR-0001) was transfected using Lipofectamine MessengerMAX (Invitrogen) at a ratio of 1:2 according to the manufacturer’s instructions. Media were changed 12 hours posttransfection, and cells were analyzed 24 hours posttransfection. Before analysis, cells were washed with 2% FBS in PBS and resuspended in 2% FBS in PBS containing DAPI for viability. Transfection efficiency was ~10%.

### Cap-dependent translation inhibition by small molecules

Stock solutions of 250 nM Torin-2 (Selleckchem, #S2817) and 25 nM rocaglamide (MedChemExpress, #HY-19356\CS 5246) were prepared with dimethyl sulfoxide (DMSO). K562 cells stably transduced with the EMCV and RUNX1-bicistronic reporters were treated with and without either drug treatment, and the MFI of GFP and mRFP was calculated 1 day later on a BD FACSAria II.

### Hair follicle immunostaining

*Translator* mice were euthanized in the second anagen (P29-P30) by CO_2_ asphyxia. Mouse dorsal skin was dissected and fresh frozen in OCT (Sakura Finetek). Fifty-micrometer sections were used for immunofluorescence staining. Slides were fixed for 10 min with 4% paraformaldehyde (PFA) in PBS at room temperature followed by extensive washes in PBS and 0.3% Tween 20 in PBS. Slides were blocked (5% donkey serum, 1% bovine serum albumin, 2% cold water fish gelatin, and 0.3% Triton in PBS) for 1 to 2 hours at room temperature, incubated with primary antibody overnight at 4°C, washed in 0.3% Tween 20 in PBS, and then incubated with secondary antibody for 4 hours at room temperature. Antibodies used: PCAD (goat, R&D AF761, 1:400) and anti-goat IgG 647-conjugated (donkey, Jackson ImmunoResearch, 705-605-147). Images were acquired using an LSM 880. For evaluating IRES/Cap, the ratio of fluorescence intensity was calculated in HFSC and TACs for GFP and mRFP using FIJI (version 2.30/1.53q). Reporter negative littermates were used to exclude background fluorescence. HFSC and TACs were identified on the basis of PCAD staining and position in hair follicle.

### Production of lentiviral particles encoding bicistronic reporters

The pEF1-EMCV reporter construct was a generous gift from S. Weinharten-Gabbay (Broad Institute). The RUNX1-IRES element was synthesized and cloned using Bsu36I and NdeI on the original pEF1-EMCV construct by Synbio (Monmouth Junction, NJ, USA). Constructs for evaluating cryptic promoter activity were generated by performing EagI-HF (NEB, #R3505) and BbsI-HF (NEB, #R3539) restriction digestion on the aforementioned constructs, followed by blunting (NEB, #E1201) and T4 DNA ligation (NEB, M0202), per the manufacturer’s instructions. Lentivirus was prepared by transfecting low-passage HEK293T with the transfer backbone, Δ8.9, and VSV-G and concentrated by ultracentrifugation of virus containing supernatants 48 to 72 hours posttransfection.

### Production of lentiviral particles encoding inducible sgPTBP1

The destination vector KOBB was generated by modification of pMJ179 (Addgene, Plasmid #85996). After digestion with Hpa1 (NEB, #R0105) and Not1 (NEB, #0189) the hU6-cloning site-scRNA sequence (table S4) was cloned into the linearized plasmid using the NEBuilder HiFi DNA Assembly Master Mix (NEB, #E2621). Nontargeting and PTBP1-targeting single guide RNA (sgRNA) sequences were generated by CRISPick (Broad Institute). The sgRNA sequences were inserted by digestion of the KOBB destination vector with BamHI (NEB, #R0136) and Xhol (NEB, #0146) and sgRNAs (table S4) followed by assembly with the NEBuilder HiFi DNA Assembly Master Mix (NEB, #E2621). Lentivirus was prepared as described in “Production of lentiviral particles encoding bicistronic reporters.” PTBP1 expression was evaluated by comparing cultured and transduced KH2/iCas9^+/−^ *Translator* mice LKS by quantitative polymerase chain reaction (qPCR). Briefly, RNA was extracted using an RNeasy Plus Micro Kit (Qiagen, #74034) and cDNA was synthesized using an iScript cDNA synthesis kit (Bio-Rad, #1708891). qPCR reactions were performed using the iTaq Universal SYBR Green Supermix (Bio-Rad, #1725122) on a CFX384 Real-Time System (Bio-Rad). The data were analyzed using the 2^−ΔΔCt^ calculations. *Beta-actin* was used as a housekeeping gene. Primers are listed in table S4.

### Bone marrow transplantations

For all transplantations CD45.1 recipient mice were lethally irradiated with 9.5–gray (Gy) total body irradiation (split dose, 12 hours apart) 16 hours before transplantation. All mice were irradiated in a pie cage (Braintree Scientific) with rotation in a JL Shepherd irradiator.

For Mx-cre inducible knockout of PTBP1, LKS sorted from WT (C57BL/6J) lineage-depleted BM or thawed PTBP1^fl/fl^ and Mx-Cre PTBP1^fl/fl^ BM generously gifted by B. Porse (University of Copenhagen) were cultured and transduced with lentiviral reporters encoding the pEF1-EMCV bicistronic reporter at a multiplicity of infection (MOI) of 20. For Cas9-inducible knockout of PTBP1, LKS sorted from KH2/iCas9^+/−^ *Translator* mice were transduced with lentivirus encoding KOBB-sgPTBP1 or KOBB-sgNT (see “Production of lentiviral particles encoding inducible sgPTBP1”). Sixteen hours posttransduction at an MOI of 20, cells were washed with 2% FBS in PBS and resuspended PBS. A total of 20,000 transduced LKS were injected retro-orbitally into recipient mice with 200,000 CD45.1 Sca1-depleted marrow, collected using the Anti-Sca1 MicroBead Kit (#130-123-14, Miltenyi Biotec).

Cas9 activation was induced by three intraperitoneal injections of doxycycline (50 μg/g; Sigma-Aldrich, #D3072) every other day in parallel with administration of water containing doxycycline (2 mg/ml) and sucrose (10 mg/ml) for 10 days. Lineage-depleted BM was analyzed 4 months posttransplantation. PTBP1^fl/fl^ and Mx-Cre PTBP1^fl/fl^ HSPCs were identified on the basis of GFP/mRFP+ expression, whereas KH2/iCas9-PTBP1^−/−^ HSPCs were identified on the basis of BFP+ (which ranged from ~10 to 25%, depending on the immunophenotype). HSPCs were characterized on the basis of immunophenotyping using the panel of antibodies outlined in “Flow cytometry analysis and sorting,” except for iCAS9-PTBP1^−/−^ HSPCs, which were analyzed with CD105 PE-Cy7 and CD150 BV650 used in place of CD150 PE-Cy7.

For primary competitive transplantations, 200,000 CD45.1 whole BM competitor cells were retro-orbitally coinjected with 20 CD45.2 SLAM-LKS sorted from the *Translator* mouse based on the IRES/Cap ratio (low, mid, and high) into lethally irradiated CD45.1 recipient mice. Every 4 weeks posttransplantation, peripheral blood was obtained by retro-orbital bleeding using heparinized microhematocrit capillary tubes (#22-362-566, Fisherbrand) and depleted of RBCs by ACK lysis (#18-156-721, Quality Biological). Samples were blocked with CD16/32 Fc (#553142, BD Biosciences) for 15 min at 4°C and then stained with CD19-BV785 (Clone 6D5, #115543, BioLegend), CD45.2-APC (Clone 104, #109814, BioLegend), CD3ε-BUV737 (Clone 145-2C11, #612771, BD), CD45.1-BV421 (Clone A20, #110732, BioLegend), Ly-6G-PE-Cy7 (Clone 1A8, #127618, BioLegend), CD8a-BV570 (Clone 53-6.7, #100739, BioLegend), CD4-AF700 (Clone GK1.5, #100430, BioLegend), B220-BV785 (Clone RA3-6B2, #103246, BioLegend), and CD11b-BV711 (Clone M1/70, #101242, BioLegend). Cells were analyzed using a BD FACSAria II (BD). For secondary BM transplantations, 1 million total BM cells were collected from primary recipients (16 weeks posttransplantation) and transplanted into lethally irradiated CD45.1 recipients.

### Cord blood transplantations

Human cord blood was purchased from the Pasquarello Tissue Bank (Dana Farber Cancer Institute). CD34+ cells were purified using the EasySep Human Cord Blood CD34 Positive Selection Kit II (#17896, Stem Cell Technologies), as per the manufacturer’s instructions. CD34+ cells were seeded at the concentration of 5 × 10^5^ cells/ml in serum-free StemSpan medium (#09650, StemCell Technologies) supplemented with penicillin (#15140-122, Gibco, 100 IU/ml), streptomycin (#15140-122, Gibco, 100 IU/ml), glutamine (#25030081, Gibco, 2 mM), SR-1 (#1967-1, Biovision, 1 μM), UM729 (#72332, Stem Cell Technologies, 500 nM), hSCF (#300-07, PeproTech, 100 ng/ml), hFlt3-L (#300-19, 100 ng/ml), hTPO (#300-18, PeproTech, 20 ng/ml), and hIL-6 (#200-06, PeproTech, 20 ng/ml) in a 5% O_2_ and 5% CO_2_ humidified atmosphere at 37°C. After 24 hours of stimulation, cells were transduced with lentiviral particles for bicistronic reporters at an MOI of 20 overnight. A total of 300,000 CD34+ cells were washed with PBS and 2% FBS, resuspended in PBS and injected intravenously into 1.5-Gy sublethally irradiated NSG mice. Lineage-depleted BM was analyzed 4 months posttransplantation.

### Caloric restriction and fasting

For 30 days, translator mice were fed ad libitum either custom control diet (Research Diets, A11051302B) containing 12% protein, 10% fat, and 78% carbohydrates, which was poorly consumed compared to animal facility chow (Prolab IsoPro RMH 3000, LabDiet, 5P75) containing 26% protein, 14% fat, and 60% carbohydrates. Mice were weighed twice per week and monitored for malnourishment. For fasting experiments, animals were restricted from eating for 24, 48, or 72 hours before euthanasia and HSPC analysis by flow cytometry, as described in “Flow cytometry analysis and sorting.” Peripheral blood analysis was performed on an Element HT5 (Heska) from retro-orbital bleeding, as described in “Generation of the *Translator* mouse.”

### Colony-forming assays

To compare clonogenicity based on IRES/Cap, equal numbers of murine cells (SLAM-LKS: 65; GMP: 165) were sorted on the basis of the ratio and reconstituted in MethoCult M3434 (#03434, Stem Cell Technologies), as per the manufacturer’s instructions. To compare clonogenicity of WT and *Translator* marrow, 20,000 total BM cells were reconstituted in MethoCult M3434 (#03434, Stem Cell Technologies), as per the manufacturer’s instructions. Colonies were enumerated by visualization 10 days postseeding. For secondary colony assays, 5000 cells isolated from primary colony assays were plated in MethoCult M3434 (#03434, Stem Cell Technologies), and colonies were enumerated by visualization 10 days postseeding. For human cultures, 1000 CD34+CD90+EPCR+CD45RA− cultured cord blood cells (transduced 4 days prior with lentiviral reporters) were sorted on the basis of the ratio of IRES/Cap and reconstituted in MethoCult H4434 (#04434, Stem Cell Technologies). Colonies were enumerated by visualization 10 days postseeding. Colony size was measured by ImageJ (NIH) on images taken by a BX41 phase contrast microscope (Olympus) using an Infinity2 microscopy camera (Lumenera).

### Smart-Seq IV RNA-seq

To compare the transcriptome of HSPCs based on their relative use of IRES/Cap, 100 SLAM-LKS, GMP, or MEP per well were sorted on the basis of IRES/Cap from the *Translator* mouse into 2.6 μl of Lysis Buffer (Takara Bio USA Inc.) followed by snap freezing at −80°C in preparation for cDNA synthesis using the SMART-Seq v4 assay. The SMART-Seq v4 assay uses the SMART technology, switching mechanism at the 5′ end of the RNA template, to generate full-length cDNA using 11 cycles. The prepared cDNA was assessed for concentration using the Quant-iT Picogreen dsDNA assay kit (Invitrogen, P7589) on the SpectraMax i3 Multi-Mode Detection Platform (Molecular Devices) and normalized to 0.2 ng/μl before library preparation. Full-length cDNA was fragmented using the Nextera technology in which DNA is simultaneously tagged and fragmented. Tagmented samples were enriched and indexed using 12 cycles of amplification with PCR primers, which included dual 8-bp index sequences to allow for multiplexing (Nextera XT Index Kit). Excess PCR reagents were removed through magnetic bead–based cleanup using PCRClean DX beads (Aline Biosciences) on a Biomek FXP Single Arm System with Span-8 Pipettor (Beckman Coulter, A31843). Resulting libraries were assessed using a 4200 TapeStation (Agilent Technologies) and quantified by qPCR (Roche Sequencing). Libraries were pooled and sequenced on the Illumina NextSeq500 high-output flow cell using paired-end 75-bp reads. Libraries were prepared using the MANTIS Liquid Handler (Formulatrix) and the Biomek FXP Single Arm System with Span-8 Pipettor (Beckman Coulter, A31843). Full-length cDNA was prepared using the SMART-Seq v4 Ultra Low Input RNA Kit for Sequencing (Takara Bio USA Inc.), and sequencing libraries were prepared using the Nextera XT DNA Library Preparation Kit (Illumina).

### Smart-Seq IV data analysis

Reads were mapped to reference genome mm10 with ENSEMBL annotation using STAR version 2.5.4a (*67*, *68*). Read counts for individual genes were produced using the unstranded count feature in HTSeq 0.9.1 (*68*). Differential expression analysis was performed using the EdgeR package after normalizing read counts and including only those genes with count per million reads (CPM) greater than 1 for one or more samples (*69*). DEGs were defined on the basis of the criteria of minimum twofold change in expression value and false discovery rate (FDR) less than 0.05 (table S2). DEGs with logFC (log fold change) greater than 1 or less than −1 with *P* values lower than 0.05 were analyzed for MSigDB Hallmark 2020 using Enrichr (*70*–*72*). Splicing of the reporter was ruled out using the FRASER R package, as previously described (*73*) (table S1).

### Polyvinyl alcohol SLAM-LKS expansion cultures

SLAM-LKS from lineage-depleted *Translator* mouse BM were sorted on the basis of the ratio of IRES/Cap as single cells and cultured as described before (*74*). Briefly, polyvinyl alcohol (PVA) expansion media were prepared with F-12 medium (Gibco) supplemented with 10 mM Hepes (Gibco), 1× PSG (penicillin-streptomycin-glutamine; Gibco), 1× ITSX (insulin-transferrin-selenium-ethanolamine, Gibco), PVA (1 mg/ml; 87 to 90% hydrolyzed; Sigma-Aldrich), AF-TPO (100 ng/ml; recombinant animal-free murine thrombopoietin, PeproTech, AF-315-14), and AF-SCF (10 ng/ml; recombinant animal-free murine stem cell factor, PeproTech, AF-250-03). Twenty cells were indexed sorted into a 96-well plate precoated with human fibronectin (EMD Millipore) using BD FACSAria II and a 100-μm nozzle. After 3 and 6 days of culture, wells were imaged using a BX41 phase contrast microscope (Olympus) using an Infinity2 microscopy camera (Lumenera). Wells containing megakaryocytes were enumerated visually.

### Generation of CRISPRi cell lines

K562 leukemic cells were first transduced with a lentiviral vector expressing dCas9-BFP-KRAB (Addgene, #135448) and purified for BFP+ cells by flow cytometry. Next, cells were transduced with the bicistronic fluorescent reporter that reads out EMCV-IRES/Cap, and BFP+mRFP+GFP+ cells were sorted as single cells into a 96-well plate for optimal clone selection using a BD FACSAria II. The activity of CRISPRi clone was validated by transducing with positive control sgRNAs targeting ST3GAL3 and SEL1L followed by qPCR analysis of gene expression. sgRNA targeting Gal4 was used as a control.

### Amplification of CRISPRi pooled libraries

Human genome-wide CRISPRi-v2 libraries (Addgene, #83969) were obtained from Addgene. Pooled libraries (~200 ng) were transformed into MegaX DH10B cells (Thermo Fisher Scientific, C640003) by electroporation (setting: 2.0 kV, 200 Ω, and 25 μF in a 0.1-cm cuvette) to achieve a transformation efficiency of >1000 colonies per sgRNA. Colonies were grown on LB agar plates overnight and harvested on the next day, followed by plasmid extraction using the NucleoBond Xtra Maxi kit (Macherey-Nagel, 740424). Next, the sgRNA target regions from the amplified libraries were PCR amplified using primers with Illumina adaptors and sequenced by next-generation sequencing to determine the sgRNA distribution of libraries.

### Genome-wide CRISPRi screen

The CRISPRi-v2 libraries were transduced in triplicate into K562 CRISPRi cells in T175 flasks at the MOI of ~0.3 (virus volume was determined by conducting a small-scale pilot test). Two days after transduction, cells were selected by puromycin (1 μg/ml) for 5 days. Then, ~2 × 10^7^ cells from low, mid, and high IRES/Cap gates were harvested from each triplicate using a BD FACSAria II. Genomic DNA was isolated using the NucleoSpin Blood Maxi kit (Macherey-Nagel, 740950). Next, sgRNA target regions were PCR amplified with Illumina adaptors and indexes using Ultra II Q5 (NEB Biolabs, M0544X) and products were purified with DNA Clean & Concentrator-100 (Zymo Research, D4030). sgRNA target regions (260 to 280 bp) were further enriched by gel extraction (Qiagen, 28704) after running agarose gel. Purified PCR products were measured by Qubit and analyzed by TapeStation before sending for next-generation sequencing on Illumina NextSeq 2000.

### CRISPRi screen data analysis

The CRISPR screening data are processed and analyzed using the MAGeCK algorithms (table S3). Raw sequencing data are preprocessed by using MAGeCK to obtain the read counts for each sgRNA, and then MAGeCK robust rank aggregation (RRA) method is used to identify negatively and positively selected genes. Briefly, MAGeCK constructed a linear model to compute the variance of gRNA read counts and assessed the difference in gRNA abundance between low and high IRES/Cap subpopulations. The selection of genes is evaluated from the rankings of gRNAs (by their *P* values) using the α-RRA algorithm. For each gene, α-RRA assigned *P* values for both positive and negative selection. Genes with significant gRNAs were analyzed with Enrichr for KEGG terms (*70*–*72*).

### Quantification and statistical analysis

GraphPad PRISM 10 was used to plot data and run statistical analysis. Sample sizes were based on prior similar works without the use of additional statistical estimations. All measurements were performed on independent biological replicates unless indicated otherwise.
